# Association between *PPAR-γ2* gene polymorphisms and diabetic retinopathy risk: a meta-analysis

**DOI:** 10.18632/aging.202433

**Published:** 2021-02-01

**Authors:** Xue-Feng Li, Guang-Bin Jiang, Shi-Yan Cheng, Ya-Feng Song, Cai Deng, Yu-Ming Niu, Jun-Wei Cai

**Affiliations:** 1 Department of Endocrinology, Taihe Hospital, Hubei Key Laboratory of Embryonic Stem Cell Research, Hubei University of Medicine, Shiyan 442000, China; 2 Department of Radiology, Suizhou Hospital, Hubei University of Medicine, Suizhou Central Hospital, Suizhou 441300, China; 3 Department of Respiratory Medicine, Suizhou Hospital, Hubei University of Medicine, Suizhou Central Hospital, Suizhou 441300, China; 4 The Personnel Section, Taihe Hospital, Hubei University of Medicine, Shiyan 442000, China; 5 Department of Stomatology, Evidence-Based Medicine and Clinical Research, Taihe Hospital, Hubei Key Laboratory of Embryonic Stem Cell Research, Hubei University of Medicine, Shiyan 442000, P.R. China

**Keywords:** PPAR-γ2, diabetic retinopathy, polymorphism, meta-analysis

## Abstract

A close association between peroxisome proliferator-activated receptor-γ2 (PPAR-γ2) and the development of diabetic retinopathy (DR) has been previously suggested. Herein, a meta-analysis was conducted to explore the association between *PPAR-γ2* polymorphisms and DR risk by performing a systematic search and quantitative analysis. Overall, fourteen articles involving 10,527 subjects were included. The pooled results did not reveal an association between *PPAR-γ2* rs1801282 C/G and DR susceptibility in the overall population (e.g., the dominant model: CG+GG vs. CC, OR=0.85, 95% CI=0.69-1.06, P=0.15, I^2^=62.9%). Furthermore, heterogeneity tests, cumulative analyses, sensitivity analyses, and publication bias analyses were conducted and showed that the results were robust. Similarly, race-based subgroup analyses and other subgroup analyses did not reveal an association between the rs1801282 C/G and DR susceptibility. In addition, no significant association was observed between *PPAR-γ2* rs3856806 C/T polymorphism and DR risk (e.g., the dominant model: CT+TT vs. CC, OR=1.12, 95%CI=0.91-1.37, P=0.28, I^2^=27.0%). Overall, based on the current sample size and the level of evidence presented in the study, the results suggest that *PPAR-γ2* gene polymorphisms are not associated with DR risk.

## INTRODUCTION

Diabetic retinopathy (DR) is the most common microvascular complication of diabetes mellitus (DM), which originates from the damage of capillary endothelial cells in DM, leads to the exudation of liquid components from the vessels into the tissues, and then causes retinopathy and dysfunction. DR is characterized by a series of typical pathological changes, including retinal detachment, vitreous hemorrhage, retinal neovascularization, neovascular glaucoma, and recurrent proliferative retinopathy, which are the major causes of sight loss and blindness in working-age adults worldwide [1, 2]. Between 2005 and 2008, the estimated prevalence of DR and vision-threatening DR in adults with diabetes in the US were 28.5% and 4.4%, respectively [3]. In China, the pooled prevalence of DR was 18.45% in diabetes and 1.14% in the general population [4]. Moreover, the number of cases of DM was estimated to increase by 150% between 2015 and 2040, and one-third of these patients may suffer from DR [5, 6]. As a prevalent diabetes-related disease, DR is catastrophic for individuals and causes severe psychological and financial burdens for families and societies not only in the US but also in developing countries [7].

The etiology and pathogenesis of DR have been studied extensively, but the underlying mechanisms remain unclear. Chronic exposure to hyperglycemia is one of the most common risk factors that ultimately results in microvascular damage and retinal dysfunction coupled with a series of biochemical and physiological changes [8]. Other factors such as hypertension, unhealthy life habits, and racial differences have also been suggested to be associated with the development of DR [9, 10]. Moreover, emerging evidence supports a strong genetic component in the etiopathogenesis of DR, including gene mutations and abnormal expression [11–13].

Peroxisome proliferator-activated receptor-γ (PPAR-γ) belongs to the nuclear receptor superfamily, which mainly mediates ligand-dependent transcriptional regulation [14, 15]. PPAR-γ plays a key role in the regulation of adipocyte differentiation, lipid metabolism, and insulin sensitivity *in vivo*. PPAR-γ comprises four functional isoforms with alternatively spliced mRNAs. PPAR-γ2 is highly expressed in adipocytes and is the key fat-selective PPAR subtype [16]. Increasing evidence shows that PPAR-γ2 may regulate inflammatory response by promoting the differentiation and activation of monocytes and macrophages and by the inhibiting the expression of inflammatory cytokines [17]. The retina is highly prone to hyperglycemia-induced molecular damage [18]. PPAR receptors, especially the γ2 subtype, mediate numerous responses related to glucose and lipid metabolism and are involved in restoring normal insulin sensitivity and glucose homeostasis [19–21]. In fact, PPAR-γ2 agonists may effectively improve retinal microcirculation and inhibit neovascularization, and these molecules have been employed in the treatment of DR [22].

The *PPAR-γ2* gene is located on chromosome 3p25 and comprises over 100 kb DNA bases including 9 exons. Rs1801282 C/G and rs3856806 C/T polymorphisms are the most widely studied loci of *PPAR-γ2*. These polymorphisms have been extensively studied in different ethnic groups and have been found to be associated with several metabolic diseases, including diabetes and the associated complications. In 1999, Ringel et al. conducted the first case-control study on the association between rs1801282 C/G and DR risk in the German population, and did not report any significant relationship with DR risk [23]. Malecki et al. reported that the rs1801282 C/G mutation had an apparently protective effect against DR in Polish individuals [24]. Moreover, the results of subsequent studies on the association of these SNPs with DR risk remain inconsistent. Therefore, we conducted a meta-analysis to further elucidate the association between *PPAR-γ2* polymorphisms and DR risk.

## RESULTS

### Study characteristics

**Figure 1:**
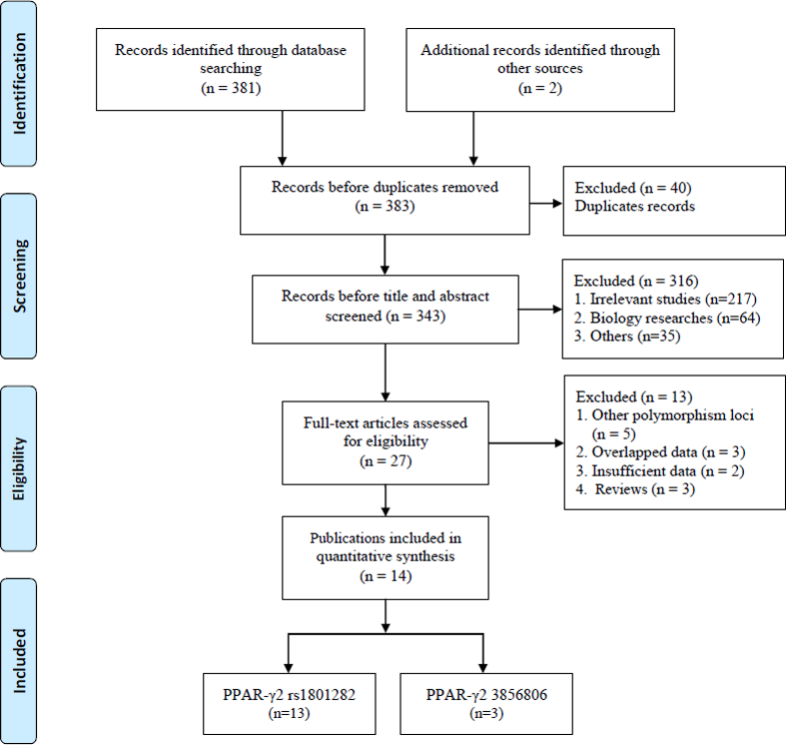
**Flow diagram of the study selection process.**

The systematic search initially yielded 383 relevant studies. The selection process is shown in Figure 1. Based on the inclusion criteria, 40 duplicate studies were excluded, 316 studies were removed after screening the titles and abstracts, and 13 additional studies were eliminated due to insufficient data and other reasons. Finally, 14 publications involving a total of 4,616 patients and 5,911 controls were included [23–36]. Among the selected studies, two common SNPs were observed: thirteen and three studies focused on the rs1801282 C/G [23–34, 36] and rs3856806 C/T polymorphisms, respectively [30, 34, 35]. The subjects in these studies were from European and Asian countries, including Germany, Poland, Italy, China, and Japan. There were four studies with missing data in HWE assessment in rs1801282 C/G polymorphism [25, 26, 29, 31], and one study showed a deviation in HWE assessment in rs3856806 C/T polymorphism [30]. The quality scores of the included studies ranged from 6 to 9, with a median of 7 according to the modified Newcastle-Ottawa Scale (NOS) [37], which ranges from 1 to 11 points and includes six dimensions: representativeness of cases; source of controls; Hardy-Weinberg equilibrium in controls; genotyping examination; subjects’ size; and association assessment (Table 1). Five studies [28, 32, 34–36] were considered high-quality, while the others [23–27, 29–31, 33] were of low quality. All collected information is provided in Table 2.

**Table 1:** Scale for quality evaluation.

**Criteria**		**Score**
**Representativeness of cases**		
Consecutive/randomly selected cases with clearly defined sampling frame with time, race, quantity and defined criteria		2
Not consecutive/randomly selected case or without clearly defined sampling frame with time, race, quantity and defined criteria		1
Not described		0
**Source of controls**		
Population-based control		2
Hospital-bases or Healthy-bases		1
Not described		0
**Hardy-Weinberg equilibrium in controls**		
Hardy-Weinberg equilibrium		2
Hardy-Weinberg disequilibrium		1
Not available		0
**Genotyping examination**		
Genotyping done under “blinded” condition and repeated again		2
Genotyping done under “blinded” condition or repeated again		1
Unblinded done or not mentioned and unrepeated		0
**Subjects’ size**		
Number ≥500		1
Number <500		0
**Association assessment**		
Assess association between genotypes and DR risk with appropriate statistics and adjustment for confounders		2
Assess association between genotypes and DR risk with appropriate statistics and without adjustment for confounders		1
Inappropriate statistics used		0

**Table 2:** Characteristics of included studies on PPAR-γ2 gene polymorphisms and diabetic retinopathy risk.

**First author**	**Year**	**Country/Racial**	**Source of controls**	**Case/Control**	**Genotype distribution**							**Genotyping methods**	***P* for HWE**	**MAF**		**NOS**
					**Case**				**Control**					**Case**	**Control**	
					**rs1801282 C/G**											
					**CC**	**CG**	**GG**		**CC**	**CG**	**GG**					
Ringel	1999	Germany/Caucasian	NDR +HC	100/713	78	20	2		521	173	19	PCR-RFLP	0.31	0.12	0.15	7
Mori	2001	Japan/Asian	NDR+HC	1626/1787	1555	71 ^a^			1656	131^a^		PCR-RFLP	NA	NA	NA	6
Zietz	2002	Germany/Caucasian	NDR	196/319	160	36 ^a^			240	79 ^a^		PCR-RFLP	NA	NA	NA	6
Herrmann	2002	Germany/Caucasian	NDR	69/376	55	13	1		272	98	6	PCR-RFLP	0.40	0.11	0.15	7
Petrovic	2005	Slovenia/Caucasian	NDR	160/101	117	40	3		79	19	3	PCR-RFLP	0.18	0.14	0.12	8
Stefanski	2006	Poland/Caucasian	NDR	99/117	65	34 ^a^			90	27 ^a^		PCR-RFLP	NA	NA	NA	6
Malecki	2008	Poland/Caucasian	NDR	121/238	86	31	4		158	73	7	PCR-RFLP	0.68	0.16	0.18	7
Costa	2009	Italy/Caucasian	HC	211/254	179	32	0		222	32	0	PCR- DHPLC	0.28	0.08	0.06	7
Liu-1	2010	China/Asian	NDR	382/378	354	28 ^a^			344	34 ^a^		PCR-RFLP	NA	NA	NA	6
Tariq	2013	Pakistan/Caucasian	NDR+HC	180/393	149	31	0		298	85	10	PCR-RFLP	0.19	0.09	0.13	8
Liu-2	2013	China/Asian	NDR	60/30	44	16	0		12	16	2	PCR-RFLP	0.27	0.13	0.33	7
Zhang	2015	China/Asian	NDR	448/344	396	48	0		311	26	1	PCR-LDR	0.56	0.05	0.04	8
Kaur	2017	India/Asian	NDR	717/608	562	141	14		480	121	7	TaqMan	0.84	0.12	0.11	9
					**rs3856806 C/T**											
					**CC**	**CT**	**TT**		**CC**	**CT**	**TT**					
Costa	2009	Italy/Caucasian	HC	211/254	171	38	2		199	44	11	Applied Biosystems	<0.05	0.10	0.13	6
Zhang	2015	China/Asian	NDR	448/344	277	153	14		232	94	12	PCR-LDR	0.52	0.20	0.17	8
Wang	2015	China/Asian	NDR	247/253	125	100	22		131	97	25	Taqman	0.27	0.29	0.29	8

HWE in control.

NDR: Diabetes mellitus without retinopathy.

HC: Healthy-based control.

^a^ Data of the CG+GG genotype.

PCR-RFLP: Polymerase chain reaction-restriction fragment length polymorphism.

PCR- DHPLC: Polymerase chain reaction-denaturing high performance liquid chromatography.

PCR-LDR: Polymerase chain reaction-ligase detection reaction.

MAF: minor allele frequency.

NOS: Newcastle-Ottawa Scale.

### Quantitative and subgroup analyses

### Association between rs1801282 C/G polymorphism and DR risk

Thirteen case-control studies including 4,369 patients and 5,658 controls focused on the association between the rs1801282 C/G polymorphism and DR risk [23–34, 36]. Overall, the frequencies of the G allele ranged from 0.05 to 0.16 in cases and 0.04 to 0.33 in controls. In terms of race differences, the frequencies of the G allele ranged from 0.09 to 0.16 in DR cases and 0.06 to 0.18 in controls in the Caucasian population, and ranged from 0.05 to 0.13 in DR cases and 0.04 to 0.33 in controls in the Asian population (Table 2).

**Figure 2:**
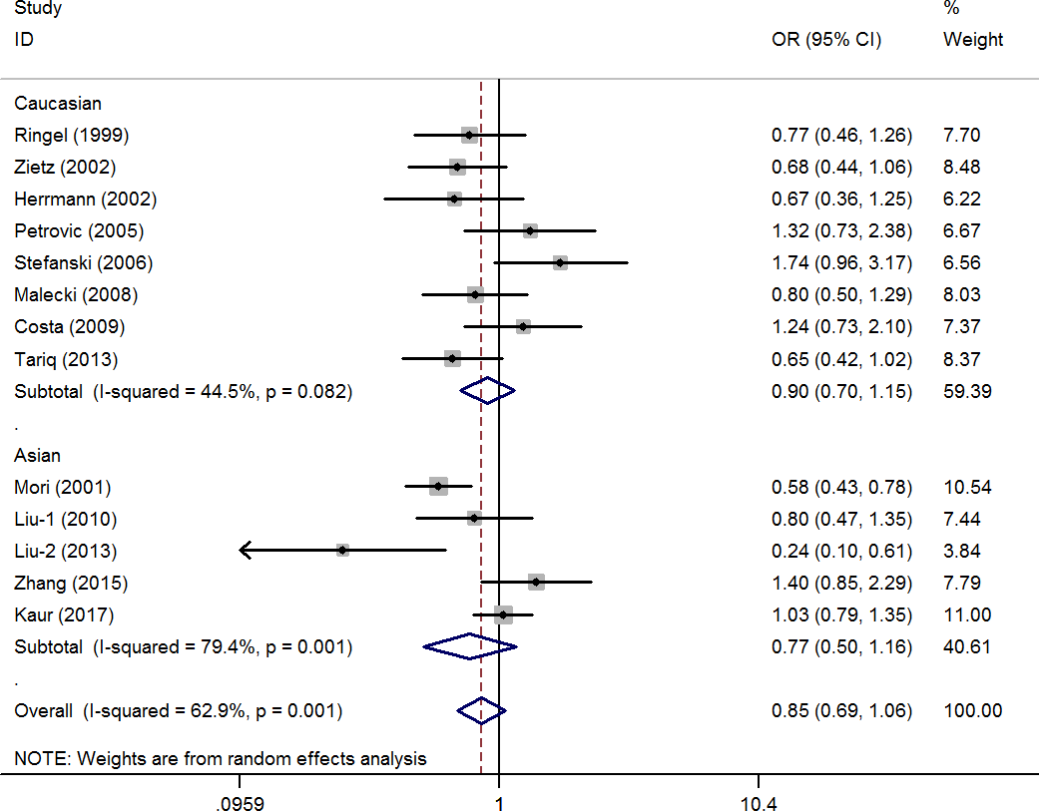
**Statistical analysis of the association between *PPAR-γ2* rs1801282 C/G polymorphism and diabetic retinopathy risk in CG+GG vs. CC model.**

The pooled results did not reveal a significant association in any of the five genetic models (G vs. C: OR=0.87, 95% CI=0.69-1.10, P=0.26, I^2^=57.8%; CG vs. CC: OR=0.90, 95% CI=0.71-1.15, P=0.40, I^2^=49.8%; GG vs. CC: OR=0.76, 95% CI=0.45-1.27, P=0.29, I^2^=17.6%; CG+GG vs. CC: OR=0.85, 95% CI=0.69-1.06, P=0.15, I^2^=62.9%, Figure 2; GG vs. CC+CG: OR=0.80, 95% CI=0.48-1.33, P=0.38, I^2^=0%) (Supplementary Table 1). Moreover, eight studies (involving 1,136 patients and 2,511 controls) and five studies (involving 3,233 patients and 3,147 controls) focused on the association between the rs1801282 C/G polymorphism and DR risk in the Caucasian population and Asian populations, respectively. There was no significant association between the rs1801282 C/G polymorphism and DR risk in either population. In addition, subgroup analyses based on the HWE status, control design, and subtype of DR revealed non-significant associations (Supplementary Table 1).

Heterogeneity was observed in allele contrast, homozygote and dominant models. Meta-regression analysis was conducted with the above-mentioned factors, and no significant factors were identified as sources of the existing heterogeneity (e.g., CG+GG vs. CC model: P=0.79 for HWE status, P=0.85 for race difference, P=0.55 for control design).

**Figure 3:**
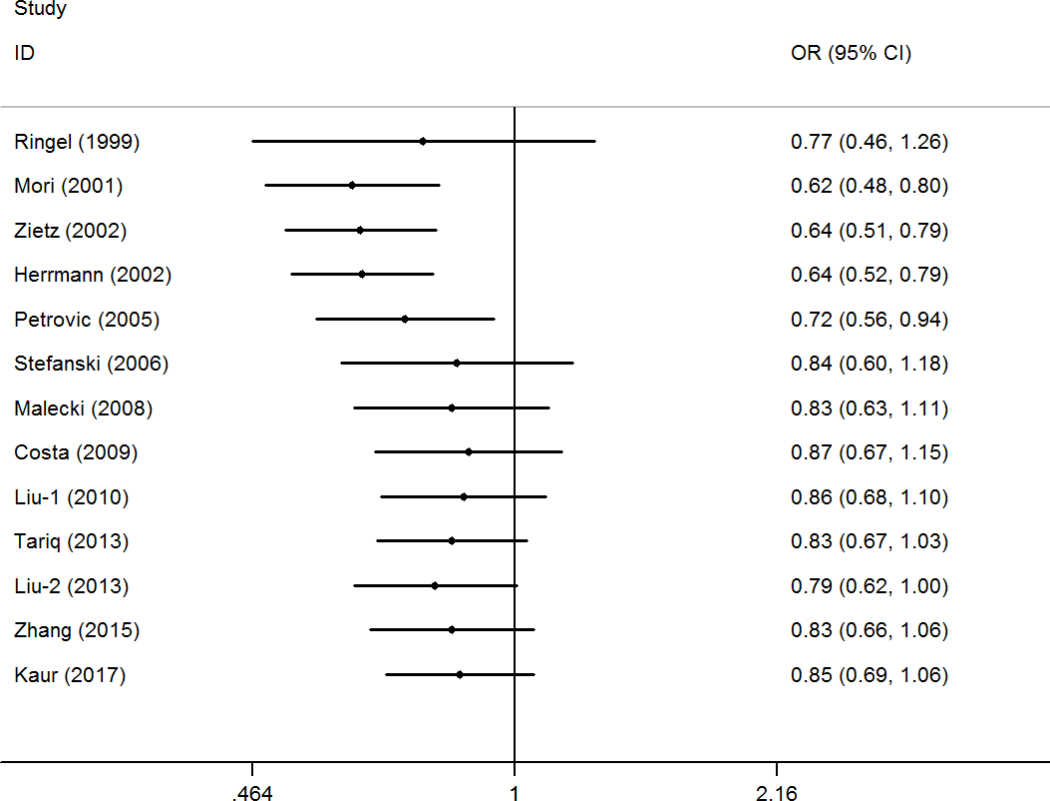
**Cumulative meta-analyses according to publication year in CG+GG vs. CC model of *PPAR-γ2* rs1801282 C/G polymorphism.**

**Figure 4:**
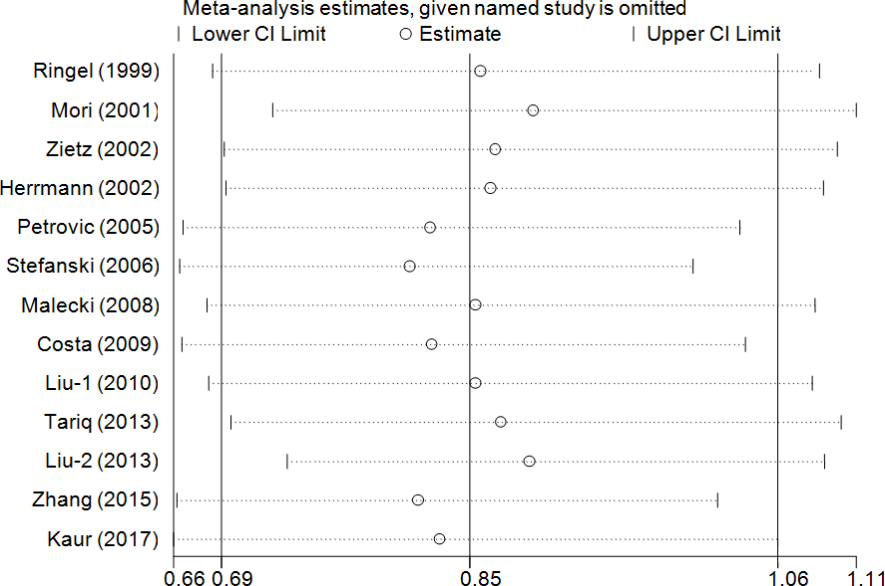
**Sensitivity analysis through deleting each study to reflect the influence of the individual dataset to the pooled ORs in CG+GG vs. CC model of *PPAR-γ2* rs1801282 C/G polymorphism.**

A cumulative meta-analysis (CMA), which aims to aggregate accumulating evidence with additional studies based on their chronological order, was performed [38]. No significant fluctuation in the results was found, indicating the stability of the results (Figure 3 for CG+GG vs. CC model). Moreover, sensitivity analysis was used to verify the stability of the results by gradually removing each study one by one and no significant change was observed (Figure 4 for CG+GG vs. CC model), indicating the credibility of the results.

**Figure 5:**
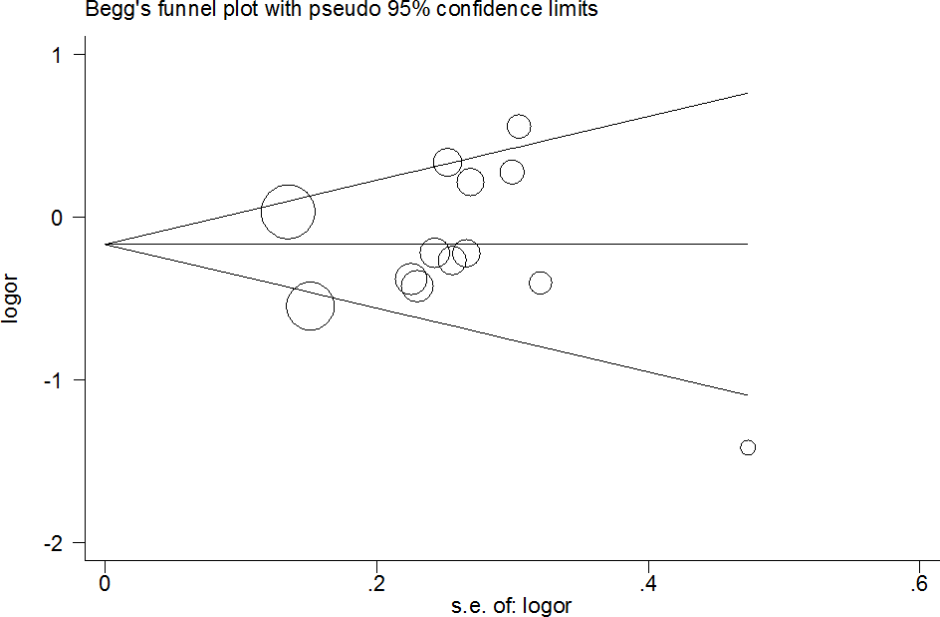
**Funnel plot analysis to detect publication bias for CG+GG vs. CC model of *PPAR-γ2* rs1801282 C/G polymorphism.** Circles represent the weight of the studies.

Publication bias was investigated, and the results revealed an obvious asymmetry in the funnel plots of homozygote and recessive models (Figure 5 for CG+GG vs. CC model). All results were confirmed using Egger’s linear regression test (G vs. C, P=0.21; CG vs. CC: P=0.32; CC vs. CC, P<0.01; CG+GG vs. CC, P=0.97; GG vs. CC+CG, P<0.01).

### Association between rs3856806 C/T polymorphism and DR risk

In addition, three case-control studies involving 902 patients and 845 controls examined the association between the rs3856806 C/T polymorphism and DR risk (Table 2) [30, 34, 35]. The frequencies of the T allele ranged from 0.10 to 0.29 in DR cases and 0.13 to 0.29 in controls (Table 2), and the pooled results indicated a non-association between the rs3856806 C/T polymorphism and DR risk (T vs. C: OR=1.01, 95% CI=0.78-1.29, P=0.11, I^2^=0%; CT vs. CC: OR=1.19, 95% CI=0.96-1.47, P=0.40, I^2^=49.8%; TT vs. CC: OR=0.75, 95% CI=0.39-1.46, P=0.40, I^2^=41.7%; CT+TT vs. CC: OR=1.12, 95% CI=0.91-1.37, P=0.28, I^2^=27.0%; TT vs. CC+CT: OR=0.74, 95% CI=0.48-1.16, P=0.19, I^2^=37.1%) (Supplementary Table 1).

## DISCUSSION

DR is one of the most serious microvascular complications of DM. DR is also a major cause of visual impairment and loss of sight in working-age populations worldwide. According to the World Health Organization (WHO), the overall prevalence of DR is almost 34.6% in patients with diabetes, and DR accounts for 4.8% of all cases of blindness worldwide, which has become an important public health issue [6, 39].

Although the mechanisms underlying DR progression have not yet been fully elucidated, hyperglycemia, hypertension, hyperlipidemia, obesity and duration of diabetes have been proven to be major risk factors for DR and have been investigated widely [40]. Insulin resistance has been previously suggested to result in hyperglycemia and is implicated in DR pathology in individuals with DM. Insulin resistance may be influenced by several factors, and the aberrant expression and/or dysfunction of PPAR may be common factors contributing to individual susceptibility to DR. *PPAR-γ2* is an isoform of PPAR-γ with an additional NH2-terminal region composed of 30 amino acids [41]. This region is considered a valuable target in the amelioration or treatment of endothelial and retinal damage due to high glucose-induced prolonged inflammation [42, 43]. PPAR-γ2 is mainly expressed in the adipose tissue; it modulates the expression of target genes involved in glucose metabolism, angiogenesis, and inflammation pathways, and is involved in the development of macrovascular and microvascular lesions [44]. Accumulating evidence show that PPAR-γ activators exert anti-inflammatory, antioxidative and anti-proliferative effects for reducing the progression of DR [45]. PPAR-γ can be expressed in the mammalian eye and is prominently present in the retinal pigmented epithelium, photoreceptor outer segments, choriocapillaris and retina. PPAR-γ ligands are potent inhibitors of corneal angiogenesis and neovascularization [46]. PPAR-γ plays an important role in the pathogenesis of DR by inhibiting diabetes-induced retinal leukostasis and leakage [47]. Animal studies have indicated that intravitreal injection of rosiglitazone or troglitazone inhibited the development of new retinal vessels [48]. Rosiglitazone delayed the onset of DR by inhibiting both the retinal leukostasis and retinal leakage in experimental diabetic rats [49]. Moreover, many studies have indicated that multiple molecular factors, including advanced glycation end products (AGEs), nuclear factor-kappa B (NF-κB), inflammatory cytokines, angiogenesis and apoptosis factors, interact with PPAR-γ to regulate the development of DR [47].

SNPs are the most prevalent forms of gene mutations and can affect the regulation of gene expression and protein activity. For *PPAR-γ2*, the rs1801282 C/G and rs3856806 C/T polymorphisms have been the most widely investigated loci and have attracted increasing attention in the past few years. Many previous studies have suggested a significant association between rs1801282 C/G and various disorders of glycolipid metabolism, such as DM, coronary heart disease and atherosclerosis.

First, Ringel et al. conducted a case-control study in 1999 and found no significant relationship between the distributions of the *PPAR-γ2* genotype and DR in the German population [23]. However, subsequent studies reported a series of inconsistent or contradictory results. Malecki et al. identified an apparently protective effect of the rs1801282 C/G mutation in Polish individuals (P=0.026 for allele contrast, P=0.014 for the dominant model and P=0.038 for the additive model) [24]. Liu et al. also reported a similar association with a decreased risk of DR in a Chinese population [33]. However, other studies did not report any significant relationship between the rs1801282 C/G polymorphism and DR risk. Such discrepancies in previous results and ambiguous relationships may be attributed to the following factors: (1) variation in nationalities and ethnicities of individuals; (2) relatively small sample sizes; (3) inconsistencies in the quality of studies with respect to the NOS evaluation; and; (4) the controls of each study consisting of healthy people or people with diabetes mellitus without retinopathy (NDR).

Therefore, we conducted a meta-analysis to explore the association between *PPAR-γ2* gene polymorphisms and DR susceptibility. To our knowledge, rs1801282 C/G polymorphism results in a missense mutation at codon 12 of the PPAR-γ2 gene, leading to the substitution of proline with alanine, which in turn leads to a conformational change in the protein structure, thereby altering the expression of genes involved in the regulation of insulin sensitivity and lipid metabolism [50]. The G allele mutation reduces the transcription level of PPAR-γ2 by decreasing the binding affinity to the cognate promoter element, thereby reducing the ability to transactivate responsive promoters [51].

Our meta-analysis of 13 trials examining rs1801282 C/G polymorphisms and DR risk is the largest and most comprehensive meta-analysis on this topic to date. No significant association between the rs1801282 C/G polymorphism and DR risk was observed, which suggests that this single polymorphic locus may not be the determining factor for DR. To explore the potential association, subgroup analysis was conducted. Eight studies on Caucasian populations and four studies on Asian populations were analyzed, and no significant difference in genotype distributions was found between in DR cases and controls. This indicated that ethnicity may not be an independent factor contributing to a positive association between the rs1801282 C/G polymorphism and DR risk. Due to the different origins of the control groups, most controls were NDR individuals, and the pooled results indicated that neither NDR individuals nor healthy controls (HCs) had a significant association with DR risk. To our knowledge, non-proliferative and proliferative DR (NPDR and PDR, respectively) are the two main subtypes of DR; the former is characterized by microaneurysms and retinal hemorrhages in the early stage followed by cotton wool spots, venous beading, and intraretinal microvascular abnormalities. PDR is a more advanced form of DR and always follows severe NPDR with the development of abnormal new retinal vessels [52]. Here, we conducted subgroup analyses of PDR and NPDR risk with respect to the rs1801282 C/G polymorphism, and no significant association was observed in the two DR subtypes. Unfortunately, the current results are based only on a limited sample size, and further studies to validate this result are required in the future.

Moreover, in the early stages of cumulative analysis, temporarily significant associations were observed between the rs1801282 C/G polymorphism and DR risk, but the final result indicated a null association when the newly published studies were added. A sensitivity analysis was performed and individual studies affected the results in the primary analysis when studies were deleted one at a time. Moreover, an obvious asymmetry was detected with Begg’s funnel plots, indicating that there was some publication bias among the included studies. This may be because the selected studies were mainly from Asia and Europe, and the languages of the papers are limited (English and Chinese). In addition, the included information is mainly published data, and other unpublished data and gray literature have not been retrieved and found at present.

In 2012, Ma et al. conducted the first meta-analysis on the association between the *PPAR-γ2* polymorphism and DR risk [53]. The authors analyzed eight case-control studies in their study and detected a protective effect in the overall population but not in Caucasian and Asian subgroups. Our study provided five additional case-control studies and a more comprehensive meta-analysis. In contrast to previous reports, no significant effect of the *PPAR-γ2* polymorphism on DR risk was observed in either the general population or stratified analysis.

Compared to previous studies, our meta-analysis provided more credible results for the following reasons: (1) a better, scientifically sound study retrieval strategy was employed; (2) larger sample sizes were collected; (3) more rigorous methodologies, such as cumulative and sensitivity analyses as well as subgroup and publication bias analyses and NOS evaluation, were conducted to ensure stability and accuracy; and (4) more subgroup analyses were conducted to explore potential associations. Despite these strengths, our study inevitably suffers from some limitations that should be addressed: (1) for each individual SNP, the number (sample size) of published studies was still limited to yield a definitive conclusion; (2) only unadjusted analyses were conducted without the original data, such as life habitat, environmental exposure, and gene-environment interactions, which limits the understanding of the underlying interaction mechanisms; (3) only studies published in English or Chinese languages were collected, which may lead to language and race biases; and (4) a moderate heterogeneity was found in some genetic models among the included studies. To our knowledge, clinical heterogeneity (clinical diversity), methodological heterogeneity (methodological diversity) and statistical heterogeneity were the three most common sources. In this meta-analysis, the HWE status, control design, and subtypes of DR may be the potential factors contributing to the existing heterogeneity. Fortunately, the heterogeneity was obviously alleviated in the stratified analysis of Caucasian populations and healthy-based controls in the rs1801282 C/G polymorphism loci.

In summary, the current evidence indicates that the *PPAR-γ2* polymorphism is not associated with DR susceptibility, despite the important role of *PPAR-γ2* in metabolic diseases. More case-control studies with larger sample sizes of groups from different ethnicities are required to verify this conclusion.

## MATERIALS AND METHODS

This meta-analysis was conducted in accordance with the guidance of the Preferred Reporting Items for Systematic Reviews and Meta-Analyses (PRISMA) statement. All collected data were extracted from published studies, and there were no ethical issues.

### Literature search

The PubMed, Embase, Web of Science, CNKI and Wanfang online databases were searched for studies examining the association between *PPAR-γ2* polymorphisms and DR susceptibility. Only studies published in English and Chinese languages were selected. The bibliographies of all included studies were reviewed to identify any other relevant studies as well. The strategy was listed (e.g., in PubMed):

#1 Peroxisome proliferator-activated receptor-γ

#2 PPAR-γ

#3 PPAR-γ2

#4 #1 OR #2 OR #3

#5 rs1801282

#6 rs3856806

#7 polymorphism

#8 variant

#9 mutation

#10 #5 OR #6 OR #7 OR #8 OR #9

#11 diabetic retinopathy

#12 DR

#13 #11 OR #12

#14 #4 AND #10 AND #13

### Inclusion and exclusion criteria

The following criteria were used to identify relevant studies: (1) case-control studies on the association between *PPAR-γ2* gene polymorphisms and DR; (2) studies with relevant genotype data in both case and control groups to evaluate the odds ratios (ORs) and 95% confidence intervals (CIs); (3) studies published only in English or Chinese; (4) the polymorphism locus was quantitatively analyzed in at least three studies;(5) when multiple publications or overlapping data were detected, the newest study or the study with the largest sample size was included. The exclusion criteria were as follows: 1) review articles, case reports and animal experiments; 2) fundamental biological research; 3) studies without enough data; and 4) duplicate or overlapping data on the same theme.

### Data extraction and quality evaluation

Two authors independently reviewed the selected studies and extracted the following information: the name of the first authors, publication date, country in which the study was performed, sample sizes of cases and controls, control design, genotyping method, frequency data for the genotype distribution in case and control groups, assessment of Hardy-Weinberg equilibrium (HWE) in the control group, and the minor allele frequency (MAF) of case and control groups. The modified NOS evaluation was used to evaluate the quality of the included studies. The scores ranged from 0 (worst) to 11 (best) points (Table 1). Studies with a score of seven points or higher indicated good research quality.

### Statistical analysis

The pooled ORs and 95% CIs were calculated to examine the statistical power of the association between *PPAR-γ2* polymorphisms and DR risk. For example, genetic models of the rs1801282 C/G locus were examined: allele contrast (G vs. C), co-dominant models (CG vs. CC and GG vs. CC), dominant model (CG+GG vs. CC), and recessive model (GG vs. CC+CG). Heterogeneity among the included studies was examined using Cochran’s Q and I^2^ tests. A fixed effects model was adopted when I^2^ ≤40%, and a random effects model was adopted otherwise. Subgroup analyses were performed according to the HWE status, ethnic differences, study country, control design, and disease subtype. Meta-regression was performed to identify the sources of heterogeneity. A cumulative meta-analysis was conducted to explore the tendency of change in the results in our meta-analysis. Sensitivity analysis was used to verify the stability of the results by gradually removing each study one by one. Potential publication bias was detected using Egger’s linear regression test and Begg’s funnel plots. All statistical analyses were performed using STATA version 14.0 (Stata Corporation, College Station, TX, USA). Statistical significance was set at p<0.05 (two-sided).

## Supplementary Material

Supplementary Table 1
