# Supplementary material for: Association between PPAR-γ2 gene polymorphisms and diabetic retinopathy risk: a meta-analysis
Source: Aging (Albany NY). 2021 Feb 1;13(4):5136–49. doi: 10.18632/aging.202433 (PMC7950267; doi:10.18632/aging.202433)
Supplement: Supplementary Table 1 [file aging-13-202433-s001.docx]

**Supplementary Table 1. Summary ORs and 95% CI of PPAR-γ2 gene polymorphisms and diabetic retinopathy risk.**

| Locus | N* | case | control | OR | 95% CI | *P* | *I*^2^ (%)^a^ |  | OR | 95% CI | *P* | *I*^2^ (%)^a^ |  | OR | 95% CI | *P* | *I*^2^ (%)^a^ | |  | OR | 95% CI | *P* | *I*^2^ (%)^a^ |  | OR | 95% CI | *P* | *I*^2^ (%)^a^ |
| --- | --- | --- | --- | --- | --- | --- | --- | --- | --- | --- | --- | --- | --- | --- | --- | --- | --- | --- | --- | --- | --- | --- | --- | --- | --- | --- | --- | --- |
| rs1801282 C/G |  |  |  | G vs. C | | | |  | CG vs. CC | | | |  | GG vs. CC | | | | |  | CG+GG vs. CC | | | |  | GG vs. CC+CG | | | |
| Total | 13 | 4369 | 5658 | 0.87 | 0.69-1.10 | 0.26 | 57.8 |  | 0.90 | 0.71-1.15 | 0.40 | 49.8 |  | 0.76 | 0.45-1.27 | 0.29 | 17.6 | |  | 0.85 | 0.69-1.06 | 0.15 | 62.9 |  | 0.80 | 0.48-1.33 | 0.38 | 0 |
| HWE-yes | 9 | 2066 | 3057 | 0.87 | 0.69-1.10 | 0.26 | 57.8 |  | 0.90 | 0.71-1.15 | 0.40 | 49.8 |  | 0.76 | 0.45-1.27 | 0.29 | 17.6 | |  | 0.88 | 0.68-1.13 | 0.31 | 56.0 |  | 0.80 | 0.48-1.33 | 0.38 | 0 |
| Ethnicity |  |  |  |  |  |  |  |  |  |  |  |  |  |  |  |  |  | |  |  |  |  |  |  |  |  |  |  |
| Caucasian | 8 | 1136 | 2511 | 0.84 | 0.70-1.02 | 0.07 | 21.6 |  | 0.87 | 0.70-1.08 | 0.21 | 14.7 |  | 0.59 | 0.29-1.22 | 0.15 | 0 | |  | 0.90 | 0.70-1.15 | 0.39 | 44.5 |  | 0.63 | 0.31-1.28 | 0.20 | 0 |
| Asian | 5 | 3233 | 3147 | 0.83 | 0.44-1.55 | 0.56 | 81.9 |  | 0.84 | 0.44-1.61 | 0.61 | 78.8 |  | 0.44 | 0.05-3.97 | 0.92 | 61.9 | |  | 0.77 | 0.50-1.16 | 0.21 | 79.4 |  | 0.56 | 0.08-3.79 | 0.55 | 52.2 |
| Design |  |  |  |  |  |  |  |  |  |  |  |  |  |  |  |  |  | |  |  |  |  |  |  |  |  |  |  |
| NDR | 12 | 4154 | 3676 | 0.82 | 0.63-1.07 | 0.15 | 63.9 |  | 0.86 | 0.66-1.13 | 0.28 | 52.3 |  | 0.72 | 0.43-1.20 | 0.20 | 28.7 | |  | 0.85 | 0.69-1.06 | 0.14 | 55.1 |  | 0.75 | 0.45-1.25 | 0.27 | 20.5 |
| HC | 4 | 2117 | 1976 | 0.87 | 0.66-1.15 | 0.32 | 22.1 |  | 0.90 | 0.66-1.22 | 0.48 | 8.0 |  | 0.58 | 0.16-2.10 | 0.40 | 0 | |  | 0.74 | 0.51-1.09 | 0.13 | 62.7 |  | 0.60 | 0.17-2-20 | 0.44 | 0 |
| Subtype |  |  |  |  |  |  |  |  |  |  |  |  |  |  |  |  |  | |  |  |  |  |  |  |  |  |  |  |
| PDR | 4 | 391 | 880 | 0.54 | 0.21-1.42 | 0.21 | 82.2 |  | 0.60 | 0.23-1.62 | 0.32 | 79.5 |  | NA | NA | NA | NA | |  | 0.62 | 0.30-1.30 | 0.21 | 74.6 |  | NA | NA | NA | NA |
| NPDR | 4 | 489 | 880 | 0.73 | 0.36-1.47 | 0.37 | 71.8 |  | 0.88 | 0.41-1.77 | 0.68 | 67.2 |  | NA | NA | NA | NA | |  | 0.73 | 0.43-1.26 | 0.26 | 64.6 |  | NA | NA | NA | NA |
|  |  |  |  |  |  |  |  |  |  |  |  |  |  |  |  |  |  | |  |  |  |  |  |  |  |  |  |  |
| rs3856806 C/T |  |  |  | T vs. C | | | |  | CT vs. CC | | | |  | TT vs. CC | | | | |  | CT+TT vs. CC | | | |  | TT vs. CC+CT | | | |
|  | | | | | | | | | | | | | | | | | | | | | | | | | | | | |
| Total | 3 | 902 | 845 | 1.01 | 0.78-1.29 | 0.97 | 50.4 |  | 1.19 | 0.96-1.47 | 0.11 | 0 |  | 0.75 | 0.39-1.46 | 0.40 | | 41.7 |  | 1.12 | 0.91-1.37 | 0.28 | 27.0 |  | 0.74 | 0.48-1.16 | 0.19 | 37.1 |
| HWE-yes | 2 | 691 | 591 | 1.11 | 0.92-1.34  34 | 0.28 | 0.0 |  | 1.24 | 0.98-1.57 | 0.08 | 0 |  | 0.94 | 0.58-1.54 | 0.81 | | 0 |  | 1.20 | 0.95-1.50 | 0.12 | 0 |  | 0.89 | 0.55-1.43 | 0.63 | 0 |
| Ethnicity |  |  |  |  |  |  |  |  |  |  |  |  |  |  |  |  | |  |  |  |  |  |  |  |  |  |  |  |
| Caucasian | 1 | 211 | 254 | 0.74 | 0.49-1.12 | 0.15 | NA |  | 1.01 | 0.62-1.62 | 0.98 | NA |  | 0.21 | 0.05-0.97 | 0.05 | | NA |  | 0.85 | 0.54-1.34 | 0.47 | NA |  | 0.21 | 0.05-0.97 | NA | 0.21 |
| Asian | 2 | 691 | 591 | 1.11 | 0.92-1.34  34 | 0.28 | 0.0 |  | 1.24 | 0.98-1.57 | 0.08 | 0 |  | 0.94 | 0.58-1.54 | 0.81 | | 0 |  | 1.20 | 0.95-1.50 | 0.12 | 0 |  | 0.89 | 0.55-1.43 | 0.63 | 0 |

^*^ Numbers of comparisons

^a^ Test for heterogeneity

ME: Middle East; EA: East Asia

NDR: diabetes without retinopathy

HC: Healthy-based control

PDR: proliferative diabetic retinopathy

NPDR: non-proliferative diabetic retinopathy
